# A random walk-based method to identify driver genes by integrating the subcellular localization and variation frequency into bipartite graph

**DOI:** 10.1186/s12859-019-2847-9

**Published:** 2019-05-14

**Authors:** Junrong Song, Wei Peng, Feng Wang

**Affiliations:** Faculty of Management and Economics/Computer center/Faculty of Information Engineering and Automation/Technology Application Key Lab of Yunnan Province, Kunming University of Science and Technology, Lianhua Road, 650050 Kunming, People’s Republic of China

**Keywords:** Driver genes, Random walk, Subcellular localization, Variation frequency, Dysregulated genes, Genomic expression

## Abstract

**Background:**

Cancer as a worldwide problem is driven by genomic alterations. With the advent of high-throughput sequencing technology, a huge amount of genomic data generates at every second which offer many valuable cancer information and meanwhile throw a big challenge to those investigators. As the major characteristic of cancer is heterogeneity and most of alterations are supposed to be useless passenger mutations that make no contribution to the cancer progress. Hence, how to dig out driver genes that have effect on a selective growth advantage in tumor cells from those tremendously and noisily data is still an urgent task.

**Results:**

Considering previous network-based method ignoring some important biological properties of driver genes and the low reliability of gene interactive network, we proposed a random walk method named as Subdyquency that integrates the information of subcellular localization, variation frequency and its interaction with other dysregulated genes to improve the prediction accuracy of driver genes. We applied our model to three different cancers: lung, prostate and breast cancer. The results show our model can not only identify the well-known important driver genes but also prioritize the rare unknown driver genes. Besides, compared with other existing methods, our method can improve the precision, recall and fscore to a higher level for most of cancer types.

**Conclusions:**

The final results imply that driver genes are those prone to have higher variation frequency and impact more dysregulated genes in the common significant compartment.

**Availability:**

The source code can be obtained at https://github.com/weiba/Subdyquency.

**Electronic supplementary material:**

The online version of this article (10.1186/s12859-019-2847-9) contains supplementary material, which is available to authorized users.

## Background

Cancer as a worldwide challenge each year deprives thousands of people’s life. Previous researchers pointed out that cancer is a somatic evolutionary process characterized by the accumulation of mutations. With the development of sequence technology, several large-scale cancer projects have generated a huge amount of cancer genomic data, such as The Cancer Genome Atlas (TCGA) [1], International Cancer Genome Consortium (ICGC) [2]. The successful of those projects help us to investigate the cancer generation and development from the gene level and meanwhile provide a good opportunity and data support to the target therapies and diagnostics. However, investigators still fail to overcome cancer because it is a big challenge to distinguish the driver mutations which promote the cancer development from those passenger mutations which confer no selective advantages [3]. Recently, many computational methods have been proposed to identify driver genes based on cancer genomics data [4, 5]. Generally, these methods can be cataloged into frequency-based method and network-based method.

Frequency-based methods are those based on the assumption that driver mutations confer a selective advantage to tumor growth and they occur more frequently with respect to background mutation across a cohort of patients [6]. For example, Dees et.al. use the Background Mutation Rate (BMR) to measure the significant mutation genes that are more frequently mutated than expected by random chance [7]. Michale et al. [6] develop MutsigCV which considers the mutation frequency involving the related biological profile e.g. DNA replication timing and transcription activity. Contrast to before-mentioned methods which mainly focused on the frequently mutated genes, Tian et al. [8] provide an opposite idea (ContrastRank), assuming rare variants are more likely to have functional effect than common variants and among the rare variants the non-synonymous single nucleotide variants have the strongest impact. They think the lower probability of a gene mutated in samples the higher probability of it being a cancer driver gene. Most of frequency-based methods have one fatal shortage, although a part of driver genes is mutated at high frequencies (*>* 20%) most of cancer mutations occur at intermediate frequencies (2–20%) or lower than the expected [9]. Therefore, it seems far from enough to identify driver genes barely considering its mutated frequency.

Recently, some researchers have found that genes perform function together and form biological networks. The gene alteration within the network may cause architectural change by removing or affecting a node or its connection within the network [4]. These changes may drive the cells to a new phenotype that may results in cancer development [10, 11]. Wang et al. found cancer genes often function as a network hub which involves in many cellular processes and forms focal nodes in information exchange between many signaling pathways [12]. Based on those findings, one group of network-based methods maps the mutated genes of one patient or a cohort of patients to gene interactive network. Then some mutated subnetworks are extracted to identify driver genes. For example, HoteNet [13] applies a propagation process on the mutated gene interactive network and extracts significantly mutated subnetworks to identify driver genes. Network-Based Stratification(NBS) method [14] and Varwalker [15] firstly stratify mutated gene interactive network of each patient into subnetworks and then use a consensus method to merge all subnetworks across all samples to identify driver genes. Another group of network-based methods assume that if one alteration impacts more connected genes whose expression change obviously (dysregulated genes), the higher possibility of this gene is a driver gene. This kind of method usually uses the mRNA expression information to identify the dysregulated genes (also called outlying genes). After that, a bipartite graph is constructed, where one part consists of mutated genes and the other part consists of outlying genes, edges connect two parts according to the connections in gene interactive network. DriverNet is an exactly model which uses the bipartite graph to prioritize the driver genes that impacts the expressions of a large number of outlying genes [16]. Shi et al. [17] improve the prediction accuracy of driver genes by utilizing the diffusion algorithm on the bipartite graph of each patient so as to establish the relationship between mutated genes and its outlying genes. Based on the bipartite graph of mutated genes and outlying genes for single sample, DawnRank [18] ranks potential driver genes considering both their own expression difference and their impact on the overall differential expression of the outlying genes in the molecular interaction network. LNDriver [19] and DriverFinder [20] are also designed very similar to DriverNet, while LNDriver incorporates the DNA length to filter mutated gene at the first step and DriverFinder identifies outlying genes considering not only cancer expression distribution but also a corresponding normal expression distribution.

Network-based methods improve accuracy of predicting driver genes to some extent. However most of aforementioned network-based methods have some shortages as they excessively rely on the network. Some of the interactions in the network are not accurate which may lead to some nosily false positive data. In order to compensate it, researchers consider integrating other biological profiles to lower the ambiguity of network. For example, Intdriver incorporates the functional information of Gene Ontology (GO) similarity and interaction network by using the matrix factorization framework to prioritize the candidate driver genes [21]. Even though this, most of methods still ignore the importance of subcellular localization. Since proteins must be localized at their appropriate subcellular compartments to perform their desired functions, and protein-protein interaction (PPI) can take place only when they are in the same subcellular compartment [22, 23]. Based on this idea, Peng et al. do a statistical test and find a result that essential proteins appear more frequently in certain subcellular compartment than nonessential proteins and the compartment importance degree varies with its containing proteins’ counts [24]. Tang et al. combine the subcellular and PPI information to build a weighted network in order to find the candidate disease genes in diabetes [25]. They assume that proteins can interact with each other only if they are localized in the same compartments and develop a method to measure the connective reliability for each pair of interconnection proteins within the protein-protein interaction (PPI) network [25]. Inspired by these ideas, we considered whether or not the prediction performance of driver genes can be improved by only considering the genes that get a large number of supports from the outlying genes in the same subcellular compartments.

In order to improve the prediction performance to a higher level, in this work, we integrated above mentioned useful biological features, i.e. mutation frequency, subcellular localization, bipartite graph to develop a new model called Subdyquency. In order to efficiently combining these features together, we applied the random walk algorithm which can not only consider gene’s self-characteristic but also involve its influence in the network. We hypothesized that driver genes are determined by itself variation frequency in a cohort of patients, the dysregulated genes caused by it and reliability connections between mutated and the dysregulated genes. Compared to previous bipartite graph-based methods (e. g. DriverNet, Shi’s Diffusion algorithm and DawnRank), Subdyquency identifies driver genes by combining their biological properties and reliable gene-gene interactions. Compared with the Dawnrank and Varwalker that are also random walk-based methods, Subdyquency only considers the influence of direct neighbors in the network instead of walking to the whole network. We implemented driver genes prediction on three cancer types, including breast invasive carcinoma (breast), lung adenocarcinoma (lung) and prostate adenocarcinoma (prostate) cancer. The prediction results show Subdyquency outperforms other existing six methods (e. g. Shi’s Diffusion algorithm, DriverNet, Muffinne-max, Muffinne-sum, Intdriver, DawnRank) in terms of recall, precision and fscore. Moreover, the consequence shows the Subdyquency is prior to these methods in identifying driver genes with significant functions and some potential driver genes that are not included in benchmark dataset.

## Methods

### Overview

**Fig. 1:**
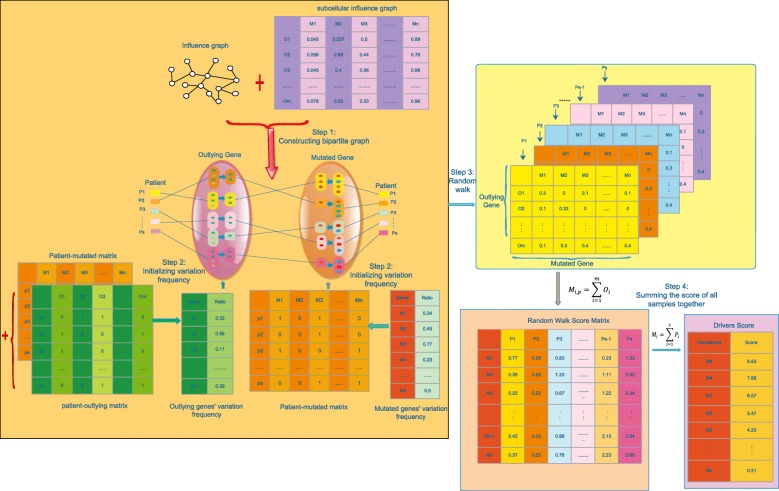
The workflow of Subdyquency. The left part with yellow background color represents the process to generate a walking score of each mutated gene for each patient. At first, we constructed the bi-partite graph between the outlying genes (dark green nodes) and mutated genes (red nodes) for each patient according to their relationship in influence graph (step 1). Each pair of interactions between mutated genes and outlying genes in bipartite graph was assigned a reliability weight according to the common subcellular compartments they belong to (top part). Then, we calculated the variation frequency for each filtered mutated genes and outlying genes as the initialized value (step 2). After random walk with three times, the walking score for each patient can be drawn (step 3). We integrated the walking score by summing up the outlying genes’ value of each mutated gene across all patients (pink background). We calculated the final score for each mutated gene by summing up its value across patients and ranked them in a descending order

We proposed a method by integrating the subcellular localization information, variation frequency, dysregulated information and influence network to prioritize the driver genes. At first, outlying genes of each patient were identified and a patient-outlying matrix was constructed according to whether or not the genes express differently in the patient. Secondly, we built the bipartite graph between the mutated genes and the outlying genes by using the patient-mutated matrix, influence graph and patient-outlying matrix. (see the details in Fig. 1). Thirdly, each pair of interactions between mutated genes and outlying genes in the bipartite graph was assigned a reliability weight according to the common subcellular compartments they belong to. Then, we calculated each mutated gene’s variation frequency and outlying gene’s variation frequency across the cohort of patients. Finally, we used the random walk algorithm initialized by the variation frequency of the mutated genes and outlying genes in a single patient and iterated three steps on the weighted bipartite graph to generate a walking score for each mutated gene in the patient. This process repeated for each patient until the random walk score matrix was generated. At last, each gene score for all patients has been summed up as its final score. We ranked mutated gene in a descending order based on their final score.

### Datasets and resources

In this research, we mainly focused on the somatic mutation and transcriptional expression data for three cancer types: lung adenocarcinoma (lung), prostate adenocarcinoma (prostate), breast invasive carcinoma (breast). Both of the somatic mutation data and transcriptional expression data were downloaded from TCGA by using R package ‘TCGA2STAT’ (https://cran.r-project.org/web/packages/TCGA2STAT/) and we only used the samples which include both of them. These three cancers were searched by using key words ‘LUAD’, ‘PRAD’ and ‘BRCA’ for lung, prostate and breast cancer, respectively. Besides, we set the searching ‘type’ parameter as the ‘somatic’ for mutation data and ‘RNASeq’ for expression data by only considering the non-silent somatic mutations and raw read counts, respectively. The downloaded TCGA somatic mutation data was represented by a binary patient-mutated matrix in which ‘1’ indicates a gene is mutated in the corresponding patient. The gene that was mutated in at least one patient was regarded as mutated gene. The expression data was prepossessed same as description in DriverNet [16]. For each patient, a gene was regarded as an outlying gene if its z-score *>* 2.0 or its z-score *<* − 2.0 according to its expression data. Furthermore, we downloaded the protein functional interaction network(2015 version) as the influence graph from Reactome database, which consists of protein-protein interactions, gene co-expression profiles, protein domain interactions, GO annotations and text-mined protein interactions [26]. The influence graph used in this work contains 12,174 proteins and 229,283 interactions. The Network of Cancer Genes (NCG4.0) which includes manually curated list of 2000 protein-coding cancer genes for 23 distinct cancer types [27] was used as the benchmark to evaluate the performance of our method. For each cancer type, Table 1 displays its sample counts, known driver gene counts in NCG4.0, mutated gene numbers, outlying gene numbers in influence graph and its density degree. For example, lung cancer dataset includes 268 known driver genes from NCG 4.0 and 230 lung cancer patients both having somatic mutation data and RNASeq data involve 5525 mutated genes, 7125 outlying genes and 54,557 weighted edges between mutated and outlying genes. In order to explain the density of network in each cancer, we used the practical edge counts to divide all edge counts (e.g.54557/7125*5525) as the density degree. The protein subcellular localization comes from the COMPARTMENTS database [28]. This database integrates evidence on protein subcellular localization from manually curated literature, high-throughput screens, automatic text mining, and sequence-based prediction methods, in which, the subcellular has been labeled as 11 different compartments, e.g. Nucleus, Golgi apparatus, Cytosol, Cytoskeleton, Peroxisome, Lysosome, Endoplasmic reticulum, Mitochondrion, Endosome, Extracellular space and Plasma membrane [25]. All of the datasets used in this research can be downloaded from the website https://github.com/weiba/Subdyquency.

**Table 1:** The datasets for each cancer type

	Lung	Breast	Prostate
Patients	230	974	331
Drivers	268	373	236
Mutated-count	5525	6510	1942
Outlying-count	7125	7915	4410
Edges	54,557	69,369	11,165
Density-degree	0. 00138591	0. 00134627	0. 00139884

The second row is the sample counts for each cancer type. The third row represents the involving driver genes for each cancer type. The Mutated count and Outlying count are the genes number for the constructed bipartite graph. Edges are the total number of the edges for each bipartite graph. Density-degree is the value of practical edges out of total edges

### Subcellular analysis

Similar to the Tang’s ideas [25], we proposed an assumption that driver genes more likely regulate their downstream gene’s expression in the same compartment and the interaction in the significance compartment is more reliability than the lower importance compartment. To support this idea, we calculated the average weighted score (details of assigning weight are in the next section) between each pair of known driver genes, outlying genes or non-driver mutated genes and outlying genes within the weighted subcellular influence graph. Result shows the higher the weight is, the more possibility of driver gene impacts outlying gene in the common significant subcellular compartment. The details for three cancers have been displayed in Table 2. The compartment coverage rate of each cancer is near to 100%, which means that all the driver genes appear at least one subcellular compartment. The average interaction weight between driver genes and outlying genes is nearly three-four times higher than the average interaction weight between general passenger mutated genes and outlying genes in lung, breast and prostate cancer. Especially for the prostate cancer, the average interaction weight between driver genes and outlying gens is more than four times higher than that between non-driver genes and outlying genes. These results sufficiently illustrate one phenomenon that most of mutated genes tend to locate in at least one compartment to perform their functions. Besides, compared with passenger genes, driver genes are more likely impact outlying genes in some significant compartments.

**Table 2:** The average weight between each pair of driver genes, outlying genes and non-driver genes outlying genes

Weight	Breast	Lung	Prostate
Compartment-coverage	0. 9878	0. 9874	0. 9778
Drivers-outlying	0. 0038	0. 0035	0. 0048
Non-drivers-outlying	0. 0011	0. 0012	0. 0011
Drivers/non-drivers	3. 455	2. 917	4. 364

The compartment-coverage is the compartment coverage of genes for each cancer type. Drivers-outlying and non-drivers-outlying are the average weight between drivers, outlying genes and non-drivers, outlying genes for the weighted subcellular bipartite graph. The last row is the value of drivers-outlying divide non-drivers-outlying

In order to verify the subcellular size information is useful in our research, we used the known cancer-related driver genes to measure the correlation between compartment size and driver genes’ counts for each cancer type. The results are shown in Table 3. It is obviously that there is a positive correlation between compartment size and the counts of known driver genes. Almost all of driver genes gather in the top three largest size compartments e.g. Nucleus, Cytosol and Plasma. Because, there are many important cell activities, like chromosome replication and transcription, that are carried in these compartments and involve in a large number of proteins [23]. Besides those largest compartments, only minority group of driver genes can be found in the ‘Endosome’ and ‘Lysosome’ with only 825 and 1960 proteins, respectively. This result suggests that the compartment size to assign weight is appropriate, since most of known driver genes likely gather in the larger size compartments.

**Table 3:** The total number of mutated genes located in each compartment

Compartment	Compartment size	Lung	Breast	Prostate
Nucleus	13,938	125	177	50
Cytosol	13,726	123	177	49
Cytoskeleton	3236	32	49	14
Peroxisome	4605	31	36	13
Lysosome	1906	17	15	4
Endoplasmic	4160	43	41	15
Golgi	3275	31	32	8
Plasma	8719	108	110	38
Endsome	825	15	13	3
Extracellular	8589	69	72	25
Mitochondrion	7130	47	50	19

The first column displays the compartment name of human. The ‘compartment size’, ‘lung’, ‘breast’ and ‘prostate’ are the total number of involving genes for each compartment

### Constructing bipartite graph

We constructed the bipartite graph according to the assumption of DriverNet that driver genes will impact on the expression of their downstream genes (dysregulate genes or outlying genes) which connect to them in the influence graph [16]. The bipartite graph consists of two parts, the right part is mutated genes denoted by M(m1,m2,m3,. ..) and the left part is outlying genes denoted by O(o1,o2,o3,.. .). The mutated genes are inferred from mutated gene profiles of all patients and the outlying genes are extracted by using the same way of DriverNet [16]. We constructed the interactions between the mutated genes and outlying genes in bipartite graph based on the rule that for each patient, the subgroup of mutated genes connects to the subgroup of outlying genes whenever each mutated gene in the functional interaction network have at least one connection to the outlying genes of another group. Specifically, In Fig. 1, red node in the mutated group represents there is at least one edge connects it to an outlying gene and the blue node means no connective edges can be found in the influence graph. Similarly, the dark green node in the outlying group means at least one edge connects it to a mutated gene and light green node means no edges connect it to a mutated gene.

### Assigning weight to bipartite graph

To compensate the error prone shortage of functional interaction network, we want to devise a method that can measure the reliability between each pair of interaction genes within the network. Since proteins can perform their functions only if they locate in appropriate subcellular compartments and protein-protein interactions happen if the proteins are in the same subcellular compartment. In this work, we use Tang’s [25] method to assign a subcellular supportive weight to the interactions between each pair of mutated and outlying gene in the constructed bipartite graph. Firstly, we measured the importance of the compartment denoted by C_*X*_ based on the number of proteins it has [23]. For each compartment, C_*X*_ divided by the largest size of compartment C_*M*_ and its final significance score SC can be calculated as follows:

1 $$ SC(I)=\frac{C_X(I)}{C_M} $$

From this formulation, the value of SC ranges from 0 to 1. *I* belongs to one of subcellular compartments, where *I ∈ {*1*,* 2*,* 3*,* 4*,* 5*......*11*}*, since there are 11 compartments in this work. The various significance scores represent the importance of different compartments, which means the compartment with larger size is more important than the compartment with smaller size, because the number of proteins involved in it is more than others. This situation implies that some interactions happen in the significant compartments should have higher score than that in other smaller size compartments. Hence, the weight assigned to each pair of related genes in the interaction network can be defined as:

2 $$ W\left(i,j\right)=\left\{\begin{array}{c}\max \left( SC(I)\right), if\ SLoc\left(i,j\right)\ne \varnothing \\ {} SC\left({C}_N\right),\kern0.5em otherwise\end{array}\right. $$

where W(i,j) is the weight between the mutated gene i and the outlying gene j. If the mutated gene i and the outlying gene j interact with each other in the same compartment (e. g. *SLoc*(*i, j*) ≠ *∅*), the interactive weight is equal to the maximum significance score of their shared compartments. Otherwise, the weight was assigned with the minimum significance score among all compartments. C_*N*_ represents the smallest size of compartment.

### Initializing variation frequency

The variation frequency of mutated genes is calculated according to the mutated genes’ abnormal times across the cohort of patients. We assume that most of driver genes are prone to mutate in many patients and impact a huge amount of down-stream genes (outlying genes) [16]. Meanwhile, the more the mutated genes impact the outlying genes that also frequently mutate across the cohort of patients, the more likely they are to be driver genes. Because previous studies found that cancer is the fact that genes act together in various signaling pathways and protein complexes [13]. If an outlying gene also frequently mutates across the cohort of patients, its connective mutated genes tend to be driver genes. Therefore, in this work, we also consider the variation frequency of outlying genes across the cohort of patients. The variation frequencies of outlying genes were calculated under two conditions. If the outlying genes also mutate in at least one patient, their variation frequencies were set according to their abnormal times across the cohort of patients. Otherwise, their variation frequencies were unified as 1 out of total sample counts. For example, the outlying gene ‘SLAMF6’ is mutated in 3 of 230 lung cancer patients. Its outlying variation frequency is 3/230. The ‘A2D1’ is outlying gene while is not mutated in any samples. Hence, its variation frequency is 1/230. At here we calculated the variation frequency of mutated gene and outlying gene based on the information of all samples. These variation frequencies were applied to the next step as the initialized score for each patient’s mutated gene and outlying gene.

### Random walk

After constructed the weighted bipartite graph, a random walk method was employed to calculate a score for each mutated gene in the bipartite graph. Given m is the number of outlying genes and n is the number of mutated genes. W is a n*m matrix. Its element w(i, j) denotes the weight of the connection between mutated gene i and outlying gene j in the weighted bipartite graph. Let Rm(i) be the ranking score of mutated gene i and Ro(j) be the ranking score of outlying gene j. M(i) denotes the variation frequency of mutated gene i (which was calculated by the last step), while O(j) is the variation frequency of outlying gene j (which was calculated by the last step). The initialized score of mutated gene and outlying gene for each patient are various according to whether it has this gene or not. Then, for each mutated gene and outlying gene in the bipartite graph, their ranking score can be computed by Formula 3 to 5. *α* is the damping factors representing the extent to which the ranking depends on the structure of the graph or itself frequency. At here, we set *α* to 0.5(details in the Result section). The result of Formula 3 was used as the input to multiply the weighted bipartite graph in Formula 4. Similarly, the result in Formula 4 would be used as the input for Formula 5. This process repeated for each patient in a given cancer. Finally, all mutated genes for each patient have a corresponding score. We added up each score across all patients as the final score of the mutated gene and ranked all of mutated genes in a descending order. The higher ranking implies the higher possibility of them to be the driver genes.

3 $$ {R}_m(i)=a\ast M(i)+\left(1-a\right)\ast \sum \limits_{j=1}^m{W}_{ij}\ast O(j) $$

4 $$ {R}_o(i)=a\ast O(j)+\left(1-a\right)\ast \sum \limits_{i=1}^n{W}_{ji}\ast {R}_m(i) $$

5 $$ {R}_m(i)=a\ast M(i)+\left(1-a\right)\ast \sum \limits_{j=1}^m{W}_{ij}\ast {R}_o(j) $$

### Assessing the performance

Similar to previous works [17–19], we evaluated the performance of our method from three aspects: prediction of known cancer genes, functional analysis, literature mining and analysis.

### Prediction of known cancer genes

We chose the top K of ranked genes as potential driver genes to evaluate the performance of our method. The accuracy of prediction depends on how well the predicted driver genes match the selected benchmarking genes(NCG 4.0), which was measured by three widely used statistical tests, i.e. precision, recall and fscore.

6 $$ Precision=\frac{TP}{TP+ FP} $$

7 $$ \kern1em Recall=\frac{TP}{TP+ FN} $$

8 $$ Fscore=2\ast \frac{Precision\ast Recall}{Precision+ Recall} $$

### Functional analysis

The somatic mutations always target the cancer genes in a group of regulatory and signaling networks to generate cancer [13, 29, 30]. Besides, those driver genes frequently occur in the functional regions of protein (such as kinase domains and binding domains) to impact the major biological functions [31]. Hence, in order to validate the efficiency of our method in distinguishing the genes sharing the most important functions and appearing some important pathways, we leveraged the DAVID database to execute GO enrichment analysis and KEGG pathway enrichment analysis. The DAVID database is a web-based analytic tool which integrates biological knowledgebase and aims at extracting biological functions from large gene/protein lists [32]. For the GO enrichment analysis, we chose the three enriched gene ontology sets COTERM_BO_DIRECT, GOTERM_CC_DIRECT and GOTERM_MF_DIRECT as the main observation objects.

### Literature mining analysis

To further prove the prediction performance of our method in distinguishing potentially unknown mutated driver genes, we leveraged one of the literature mining method(called cociter) to figure out the co-citation of the predicted driver genes with the keywords cancer type (i.e. ‘lung’, ‘breast’, ‘prostate’), ‘driver’ and ‘cancer’ [33]. The cociter is a literature mining approach which is used to evaluate the significance of co-citation for any gene set from the 8,077,952 genes in the National Center for Biotechnology Information (NCBI) Entrez gene database.

## Results

To evaluate the performance of our method, we compared our method with six existing methods, DriverNet [16], Shi’s Diffusion algorithm (namely Diffusion) [17], Muffinne-max (namely Muf_max) [34], Muffinne-sum (namely Muf_sum), Intdriver [21] and Dawn-Rank [18]. The DriverNet [16] and Shi’s Diffusion algorithm [17] are constructed based on the bipartite graph and divide the patients’ genes as mutated and outlying subgroups according to the mutated profile and expression information. Both Muf_max and Muf_sum map the mutated genes to gene functional network and leverage the variation frequency of mutated genes by considering the impact of either the most frequently mutated neighbor or all direct neighbors [34]. Intdriver combines the biological GO similarity profile with gene functional network to accumulate the accuracy of final result [21]. The DawnRank uses the random walk on the bipartite graph of mutated genes and outlying genes to identify the driver genes for specific patient [18]. We set the IntDriver turning parameters *λN*, *λS* and regularization parameter *λV* to the default value 0.3, 0.7 and 0.01 separately. The input of DawnRank requires the normal and tissue expression data for each person. But, since the limitation of downloaded datasets from TCGA, only part of patients can be found that both have the normal and cancer expression information. In this research, we found only 110, 58 and 52 samples that both have normal and tumor gene expression information for breast, lung and prostate respectively. Besides, the DawnRank’s free parameter was set to 3 according to the recommendation of authors.

All comparison methods were implemented on three types of cancers, i.e. lung, prostate, and breast cancer and evaluated from three aspects, prediction of known cancer genes, functional enrichment analysis and literature mining analysis. The result section was organized as follows. Firstly, we evaluated the effect of the parameter *α* on the performance of our method. Secondly, we compared the performance of our method with other six existing methods for each cancer type. Then, we did the frequency-based comparison of each method. Lastly, in order to verify the robustness of our method, we tested the performance by extracting samples with different sizes.

### Effects of parameter *α*

*α* in our method has been used as a trade-off to weigh the dependence degree between its own profile and the connecting network. In order to clearly illustrate the effects of *α*, we calculated the area under the Precision-Recall curve (AUC) for every cancer type under different *α* values ranging from 0 to 1, by adding 0.1 for each iteration. According to our method (mentioned in methods and materials section), setting *α* to 0 represents the final result only depending on the bipartite graph and setting *α* to 1 means the final result is only influenced by itself profile (e.g. variation frequency). AUC values for each cancer type and different *α* values are displayed in Table 4. It is clear that the result tendency for all cancer types stays in a relatively steady status with less than 0.16 gap between max and min AUC values in average. Among them, the breast and lung cancer are in a similar increasing tendency when *α* increasing from 0 to 0.7 and slightly decreasing after that. While the AUC values of the prostate cancer are almost decreasing from 0.5171 to 0.3416 when *α* ranging from 0 to 1. We supposed the reason for setting *α* to 0 achieving the prostate’s highest AUC value is that only 30 out of 126 genes mutate more than 3 patients in prostate cancer and the rest of genes seldom mutate across all patients. Hence, compared with subcellular weighted interactive network, variation frequency makes smaller impact on identification of the driver genes of prostate cancer. Besides, for the other two cancer types (e.g. lung and breast), their AUC values achieve the maximum when *α* near to the middle where incorporates itself variation frequency and the impact of network. Based on above analysis, both the variation frequency and subcellular weighted interactive network make more or less impact upon identification of the driver genes of all cancers. Besides, the AUC values increasing from 0.1 to 0.9 keep in a relatively steady status for all cancers. Hence, we chose the median value 0.5 as the static *α* value for each cancer. This setting means the subcellular weighted interactive network and variation frequency of mutated genes or outlying genes make the equal contribution to final score.

**Table 4:** Performance comparison with respect to different values

a	Breast	Prostate	Lung
0	0.4139	0.5171	0.3545
0.1	0.4129	0.5111	0.3604
0.2	0.4363	0.4706	0.3641
0.3	0.4574	0.4833	0.3894
0.4	0.4709	0.4634	0.4138
0.5	0.4771	0.4651	0.4177
0.6	0.4762	0.4281	0.437
0.7	0.4763	0.3957	0.4377
0.8	0.4672	0.385	0.4334
0.9	0.4219	0.372	0.4135
1	0.3627	0.3416	0.4204

The calculated AUC values of Subdyquency for each cancer type under different *α* values

Based on the above analysis, both the variation frequency and subcellular weighted interactive network make more or less impact upon identification of the driver genes of all cancers. Besides, the AUC values increasing from 0.1 to 0.9 keep in a relatively steady status for all cancers. Hence, we chose the median value 0.5 as the static *α* value for each cancer. This setting means the subcellular weighted interactive network and variation frequency of mutated genes or outlying genes make the equal contribution to final score.

### Result for lung cancer

Lung cancer as the top ten killer cancers occurred in 1.8 million people and leaded millions people death in 2012. In this research, we analyzed 230 lung cancer patients that both have somatic mutation data and expression information in TCGA and extracted the related subcellular bipartite graph with 5525 mutated genes, 7125 outlying genes. After applying our method, all mutated genes acquired ranking scores for each patient and the final score of mutated genes were calculated by accumulating all corresponding scores across the cohort of patients. The performance of our method was assessed by comparing it with other existing methods in the aspects of the prediction of known cancer genes and the literature mining analysis. Besides, we also did the functional enrichment analysis in pathway and GO aspects in order to prove the biological functions of the identified driver genes.

### Prediction of known cancer genes

**Fig. 2:**
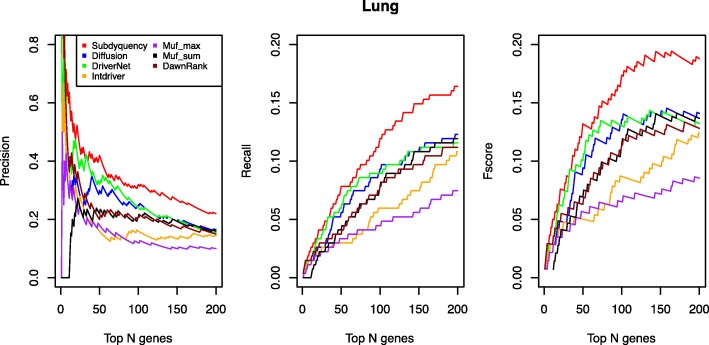
Prediction performance Comparison of each method for lung cancer in terms of Precision, Recall and Fscore values. The figure shows the comparison for lung cancer of precision, recall and fscore for top ranking genes in the seven methods. The X-axis represents the number of top-ranking genes. The Y-axis represents the score of the given metric

We selected K of genes ranked in the top list by each comparison method as candidate driver genes. According to the benchmark dataset, the fscore, recall, precision values can be calculated to evaluate the performance of each method. With difference of the values of K ranging from 1 to 200, the fscore curve, recall curve and precision curve can be drawn. Figure 2 shows that our results in total remarkably outperform other existing methods. Specifically, for our result, there are 44 out of top 200 driver genes can be found in the NCG 4.0, compared with only 16, 18, 19, 22, 25 for Muf_max, Shi’s method, Intdriver, DriverNet, Muf_sum respectively. The details of prediction of known cancer genes for lung cancer are supplied in the Additional file 1.

### Literature mining analysis

We searched the top 30 candidate driver genes together with key terms ‘cancer’, ‘driver’ and ‘lung’ in the cociter website. The higher cocitation score implicates the stronger association between the genes and the key terms.

Table 5 shows that some significant well-known genes like TP53, KRAS, EGFR, PIK3CA, ATM are showed in our top list. Although they are also identified by most of other methods, their ranking positions are not higher than ours. The well-known suppressor TP53 which disrupts the cell cycle arrest and the apoptosis pathways in human cancer ranks first in our method, 36th in Diffusion algorithm and 12th in Muf_sum. The Kirsten rat sarcoma (KRAS) is said to be one of the most activated oncogenes with 17 to 25% of all human tumors harboring an activating KRAS mutation, resulting in gene activation with transforming ability of the mutant proteins [35]. The KRAS ranks third in our list but ranked 20th in Diffusion algorithm and 102th in Muf_max. The PIK3CA is known as the regulator of cellular growth and proliferation, which ranks 14th in our method but 56th in Muf_sum, 109th in DawnRank and even cannot find in Muf_max and Intdriver. It is co-cited with ‘cancer’ for 1199 times and regarded as driver genes in 183 publications and is related to ‘lung’ 54 times. The result shows our method can not only prioritize some important genes but also can identify unknown cancer genes that are missed by the NCG 4.0. For example, the transcription factor STAT3 is constitutively activated in many human cancers and makes big contribution in modulating cancer cell proliferation, survival, metastasis and so on [36]. It was co-cited with cancer for 1824 times and was 418 times related with ‘lung’, and 27 times with ‘driver’. The CREBBP has been used as coordinating numerous transcriptional responses that are important in the processes of proliferation and differentiation [37]. It co-appeared with ‘cancer’ for 117 times, with ‘lung’ for 15 times, and with ‘driver’ for 2 times.

**Table 5:** Cociter analysis of top 30 lung cancer driver genes identified by our method

Genes	Driver	Lung	Cancer	Is_driver	Ours	Diffusion	Muf_max	Muf_sum	IntDriver	Driver Net	Dawn Rank
TP53	110	999	6772	1	1	36	5	12	5	1	1
TTN	2	1	10	1	2	2274	2	13	2	15	156
KRAS	172	1217	3525	1	3	20	102	15	19	3	2
RYR2	2	4	3	0	4	681	101	16	11	21	493
MUC16	1	31	338	1	5	1587	NA	NA	3	16	507
UBC	2	17	134	0	6	4	100	1	NA	2	NA
EGFR	166	2849	4748	1	7	2	7	22	168	4	4
SPTA1	1	2	3	0	8	140	NA	NA	24	5	13
LRP1B	2	8	17	1	9	5321	NA	18	8	482	1508
DMD	3	17	23	0	10	164	3	31	157	74	9
STK11	8	160	504	1	11	205	NA	34	70	31	14
MUC17	0	0	9	0	12	484	NA	NA	13	161	791
ANK2	0	1	4	0	13	160	NA	NA	80	62	6
PIK3CA	54	183	1199	1	14	3	NA	56	NA	6	109
ACTN2	1	3	7	0	15	27	9	40	NA	77	20
FAT3	1	2	1	0	16	330	NA	NA	48	89	224
COL11A1	1	9	21	1	17	215	NA	30	16	12	18
PCLO	1	0	4	0	18	5326	NA	NA	36	851	2664
PLCG2	1	2	13	0	19	10	NA	137	NA	34	NA
NF1	11	16	165	1	20	40	NA	108	129	19	22
PRKCB	1	11	41	1	21	6	NA	60	NA	17	NA
PCDH15	1	2	4	0	22	227	NA	19	31	11	98
STAT3	27	418	1824	0	23	7	97	45	NA	20	35
CREBBP	2	15	117	0	24	1	38	110	NA	10	112
PLCB1	1	7	9	0	25	24	NA	52	192	24	26
MYH2	1	4	3	0	26	55	NA	49	108	14	27
ATM	5	139	1377	1	27	133	12	144	NA	27	417
MYH8	1	0	0	0	28	291	8	81	NA	1157	804
ZNF536	1	0	4	1	29	1907	NA	NA	25	1039	1458
APOB	2	4	27	0	30	480	NA	25	15	43	21

The first to the fourth column show the co-appeared counts of top 30 identified genes with ‘driver’, ‘lung’ and ‘cancer’ (from the left to the right). Is_driver indicates whether the given gene is a driver or not. The left columns represent the rank positions of identified genes in Subdyquency, Diffusion, Muf_max, Muf_sum, IntDriver, DriverNet and DawnRank respectively

### Functional analysis

We used the DAVID on-line database to perform the functional and pathway enrichment analysis for the top 200 candidate driver genes of lung cancer. For the functional analysis, the chosen genes were categorized in the GOTERM_BP_FAT, GOTERM_CC_MFAT and GOTERM_MF_FAT set. In terms of biology process, the candidate driver genes play more roles in the regulation of transcription, intracellular signaling cascade, cell surface receptor linked signal transduction, cell adhesion, regulation of cell death and apoptosis cell cycle etc. (see Additional file2). With respect to the cellular component, the top 200 genes significantly enrich in the plasma membrane, intracellular non-membrane-bounded organelle, cytoskeleton, nuclear lumen, cytosol, cell fraction etc. (see Additional file 2). Finally, in the molecular function, the identified driver genes have some important functions such as the metal ion binding, nucleoside binding, ATP binding, structural molecule activity, transcription regulator activity, protein kinase activity, enzyme binding etc.(see Additional file 2). For the pathway analysis, we adopted the KEGG category and found driver genes enrich in the Focal adhesion, Regulation of actin cytoskeleton, ErbB signaling pathway, MAPK signaling pathway, Non-small cell lung cancer, Chemokine signaling pathway, Calcium signaling pathway, Wnt signaling pathway etc. which are significant associated with lung cancer (see Additional file 2).

### Results for breast cancer

In the U. S., breast cancer is the second most common cancer in women. It can occur in both men and women, but it is rare in men. At here, we focused on 974 patients that both have somatic mutation data and expression information in TCGA and extracted 6510 mutated genes and 7915 outlying genes to compose the bipartite graph.

### Prediction of known cancer genes

**Fig. 3:**
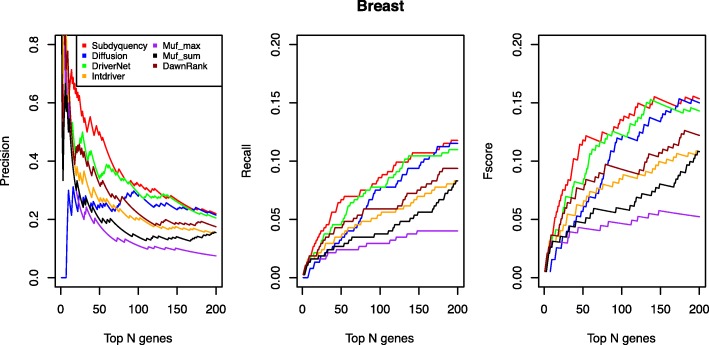
Prediction performance Comparison of each method for breast cancer in terms of Precision, Recall and Fscore values. The figure shows the comparison for breast cancer of precision, recall and fscore for top ranking genes in the seven methods. The X-axis represents the number of top-ranking genes. The Y-axis represents the score of the given metric

From the top 200 listed candidate driver genes, our method accurately identified 44 driver genes that can be found in the NCG 4.0. We supposed the most efficiency method can prioritize as many as possible driver genes in the top list. Figure 3 shows that our result was the best one to prioritize the driver genes from the top 130 listed candidate driver genes. Among those methods, the result of DriverNet is the closest one to ours. Specifically, from the top 1 to 130 genes selected as candidates, our method always acquires higher values than DriverNet in fscore, recall and precision curves while with more than top 130 genes being considered, DriverNet gradually keeps closer to us with only 0.004 less in top 150 listed genes in terms of fscore. However, when selecting the top 200 genes as candidate driver genes, our result keeps the best performance. Its fscore achieves 0.154 compared with Diffusion (0.15), Muf_max (0.052), DriverNet (0.143), DawnRank (0.122), IntDriver (0.108) and Muf_sum (0.108). The details of prediction of known cancer genes for breast cancer are supplied in the Additional file 1.

### Literature mining analysis

Table 6 shows that for breast cancer, some important driver genes in our top list can be found. Our top 6 ranked genes are very similar with DriverNet while very different with Diffusion and Muf_max. The well-known suppressor TP53 still ranks in the first position by our method, DriverNet and Muf_sum but ranks 255th by the Diffusion algorithm. The oncogene PIK3CA which is the one of most likely gain-of-function mutated in the breast cancer ranks in the second place by our method while in the 170th by Diffusion and 336th by Muf_max [38]. The putative tumor suppressor gene EP300 ranks in the 5th by our method while 73th in Diffusion, 135th in Muf_max, 175th by DawnRank and even neglectes by Intdriver. The CGH1 is key regulator adhesive properties in epithelial cells which mutates frequently in breast cancer [39]. It ranks 6th by our method, 39th by Diffusion method and 462th by Muf_max. It should be noted that some genes highly related with breast cancer rank at top but are missed by the NCG4.0 such as the CREBBP, RHOA, HDAC1, ATM and MYC. Among these genes, the ATM and MYC co-appear with item ‘cancer’ for 1377 and 1978 times, with ‘breast’ for 408 and 383 times respectively. It means our method can not only prioritize some significant driver genes but also identify some unknown driver genes.

**Table 6:** Cociter analysis of top 30 breast cancer driver genes identified by our method

Genes	Driver	Breast	Cancer	Is_driver	Ours	Diffusion	Muf_max	Muf_sum	IntDriver	Driver Net	Dawn Rank
TP53	110	1356	6772	1	1	255	2	1	2	1	2
PIK3CA	54	334	1199	1	2	170	336	6	1	2	1
UBC	2	30	134	0	3	263	224	2	NA	3	128
TTN	2	1	10	0	4	3470	554	19	3	6	104
EP300	4	86	269	1	5	73	135	4	NA	5	175
CDH1	19	358	1410	1	6	39	462	64	5	4	8
PIK3R1	7	21	131	1	7	174	354	30	NA	9	24
GATA3	8	122	154	1	8	91	382	2687	4	8	4
CREBBP	2	41	117	0	9	53	9	44	NA	7	NA
RHOA	6	100	334	0	10	213	693	141	NA	12	199
MAP3K1	2	62	135	1	11	136	1363	184	7	20	3
BRCA1	22	4017	4652	1	12	24	121	144	NA	11	NA
ERBB2	78	4332	5335	1	13	77	10	54	NA	62	79
NCOR1	3	45	109	1	14	151	31	539	30	15	52
SIN3A	3	12	49	0	15	220	93	541	NA	17	NA
ERBB3	4	178	354	1	16	78	407	139	76	27	6
HDAC1	4	99	427	0	17	104	24	128	NA	23	NA
MUC16	1	20	338	1	18	654	7281	1885	6	28	905
DMD	3	2	23	0	19	63	567	114	24	70	5
PTEN	64	672	3047	1	20	203	3	11	41	118	262
ACTB	3	14	61	0	21	2	1244	82	NA	14	131
RB1	10	124	689	1	22	210	1	5	NA	58	35
ATM	5	408	1377	0	23	17	42	423	157	45	NA
ERBB4	4	220	350	1	24	79	468	117	NA	53	217
STAT3	27	332	1824	0	25	242	43	51	NA	31	185
DYNC1H1	2	2	9	0	26	66	1395	654	53	10	17
MYC	45	383	1978	0	27	149	40	46	NA	197	NA
SP1	3	108	393	1	28	231	83	21	NA	67	NA
NEB	1	1	4	0	29	1151	1037	2188	31	400	1162
PLCG2	1	2	13	0	30	181	439	121	NA	49	NA

The first to the fourth column show the co-appeared counts of top 30 identified genes with ‘driver’, ‘breast’ and ‘cancer’ (from the left to the right). Is_driver indicates whether the given gene is a driver or not. The left columns represent the rank positions of identified genes in Subdyquency, Diffusion, Muf_max, Muf_sum, IntDriver, DriverNet and DawnRank respectively

### Functional analysis

In terms of the biology process, the top 200 potential breast driver genes mainly focus on the regulation of transcription, intracellular signaling cascade, transcription, signal transduction, regulation of cell death, regulation of apotheosis, phosphorus metabolic process etc. (see Additional file 3). In the respect of cellular component, the identified genes mainly locate in the organelle, plasma membrane, organelle lumen, nuclear lumen, cytosol, cytoskeleton, chromosome etc. (see Additional file 3). For the molecular function aspect, they enrich in the ion binding, DNA binding, transcription regulator activity, nucleotide binding, ATP binding, transcription factor activity, protein kinase activity etc. (see Additional file 3). For the pathway aspect, the candidate diver genes enrich in the breast cancer related pathway, including Focal adhesion, ErbB signaling pathway, Jak-STAT signaling pathway, Neuotrophin signaling pathway, MAPK signaling pathway etc. (see Additional file 3).

### Results for prostate cancer

It is well-known that prostate cancer is the second most common cancer among men. In this work, we focused on 331 prostate patients that both have somatic mutation data and expression information in TCGA and extracted 1942 mutated genes and 4110 outlying genes to compose the bipartite graph.

### Prediction of known cancer genes

**Fig. 4:**
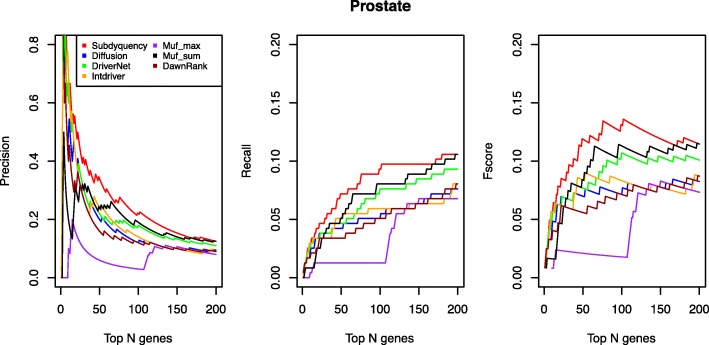
Prediction performance Comparison of each method for prostate cancer in terms of Precision, Recall and Fscore values. The figure shows the comparison for prostate cancer of precision, recall and fscore for top ranking genes in the seven methods. The X-axis represents the number of top ranking genes. The Y-axis represents the score of the given metric

From the Fig. 4, our result is obviously the best one from the beginning to the end in terms of precision, recall and fscore curves. Especially in fscore curve when selecting top 50, 100 and 150 of genes as candidate driver genes, the fscore of our method achieve the 0.119, 0.131 and 0.119 respectively, compared with the lower one Muf_sum with only 0.084, 0.113 and 0.109 on these three points. In the recall curve, the Muf_max that is the one closest to us has the recall values of 0.021, 0.012, 0.008 less than us when selecting top 50, 100 and 150 of genes as candidate driver genes. The similar situation also occurs in the precision curve. The details of prediction of known cancer genes for prostate cancer are supplied in the Additional file 1.

### Literature mining analysis

From Table 7, there are 7 driver genes in our top 10 gene list that are related with prostate cancer in NCG4.0, including TP53, SPOP, FOXA1, MUC16, ATM, CTNNB1 and SPTA1. Besides, our method also prioritizes some significant driver genes which are put in the bottom position or even neglected by other methods. For example, the tumor suppressor PTEN which is important to regulate the cell survival signaling ranks 18th by our method while 715th by Diffusion, 111th by Muf_max, 147th by DriverNet and neglectes by the DawnRank [40]. The BRAF is one of the most common mutated gene in prostate cancer which ranks in the 22th by our method while 245th by Diffusion, 35th by Muf_sum, 57th by DriverNet, 42th by DawnRank and forgets by Muf_max. The famous tumor suppressor APC which co-appears with ‘cancer’ for 2016 times, with ‘prostate’ for 59 times ranks in the 29th in our method, while 183th in IntDriver, 71th in DriverNet and is missed by DawnRank, Muf_max and Muf_sum. Besides, some genes highly related with prostate cancer that are ignored by NCG4.0 are also identified by our method. For instance, the BRAC1 is the well-known key pathogenic factor for prostate and breast cancer [41]. It co-appeared with ‘cancer’ for 4652 times, with ‘prostate’ for 156 times and 22 times for ‘driver’. The SMAD4 that is found to co-appear with ‘cancer’ for 759 times with ‘prostate’ for 41 times and 14 times for ‘drivers’ is also forgotten by NCG 4.0. Besides, the other listed genes (GLI1 and SP1) which are observed to be the highly related genes are also missed by the NCG 4.0.

**Table 7:** Cociter analysis of top 30 prostate cancer driver genes identified by our method

Genes	Driver	Prostate	Cancer	Is_driver	Ours	Diffusion	Muf_max	Muf_sum	IntDriver	Driver Net	Dawn Rank
TP53	110	298	6772	1	1	2	108	4	3	1	1
SPOP	4	24	43	1	2	1711	13	3	2	4	115
TTN	2	0	10	0	3	1713	2	2	1	25	51
FOXA1	10	69	182	1	4	10	109	17	6	5	2
MUC16	1	8	338	1	5	1712	NA	NA	4	76	184
ATM	5	61	1377	1	6	17	110	18	9	11	18
CTNNB1	44	170	2517	1	7	1	112	16	NA	2	21
OBSCN	0	0	7	0	8	1705	1	22	50	174	417
SPTA1	1	0	3	1	9	1702	NA	NA	8	15	6
MUC17	0	0	9	0	10	1710	NA	NA	16	58	4
PLCB4	3	0	4	0	11	14	NA	27	NA	13	NA
EGFR	166	144	4748	1	12	3	120	24	NA	6	NA
LRP1B	2	1	17	0	13	1681	NA	21	20	230	NA
BRCA1	22	156	4652	0	14	7	134	47	NA	7	23
FAT3	1	1	1	0	15	26	NA	NA	5	35	65
PIK3CA	54	34	1199	1	16	5	NA	31	53	9	NA
KMT2C	4	2	23	0	17	887	NA	NA	10	33	NA
PTEN	64	642	3047	1	18	715	111	25	38	147	NA
RP1	1	0	9	0	19	NA	NA	NA	31	NA	695
PIK3R2	2	5	25	0	20	4	NA	NA	NA	8	NA
UBC	2	10	134	0	21	13	28	1	NA	3	NA
BRAF	126	33	2175	1	22	245	NA	35	14	57	22
KMT2D	2	2	25	0	23	409	NA	NA	13	61	NA
SMAD4	14	41	759	0	24	6	NA	135	NA	17	42
ROCK1	2	17	150	0	25	70	NA	88	NA	64	NA
HDAC3	2	11	100	0	26	46	143	147	NA	21	NA
HSPA8	1	9	96	0	27	12	114	40	NA	14	NA
GLI1	9	41	403	0	28	9	NA	108	NA	41	54
APC	21	59	2016	1	29	11	NA	NA	183	71	NA
SP1	3	38	393	0	30	8	130	20	NA	12	NA

The first to the fourth column show the co-appeared counts of top 30 identified genes with ‘driver’, ‘prostate’ and ‘cancer’ (from the left to the right). Is_driver indicates whether the given gene is a driver gene or not. The left columns represent the rank positions of identified genes in Subdyquency, Diffusion, Muf_max, Muf_sum, IntDriver, DriverNet and DawnRank respectively

### Functional analysis

We adopted the top 200 of prostate candidate driver genes to do the enrichment analysis. The result shows, in the biology process, the identified genes enrich in the regulation of transcription, cell cycle, intracellular signaling cascade, regulation of programmed cell death, cell adhesion, regulation of apoptosis, regulation of metabolic process, homeostatic process, phosphorus metabolic process etc. (see Additional file 4). For the cellular component, they focus on the organelle, plasma membrane, organelle lumen, cytoskeleton, nuclear lumen, cytosol, cell fraction, chromosome etc. (see Additional file 4). With respect to the molecule function, they enrich in the ion binding, DNA binding, transcription regulator activity, ATP binding, transcription factor activity, nucleotide binding and so on (see Additional file 4). In the pathway enrichment analysis, the identified genes enrich in the Focal adhesion, prostate cancer, Chemokine signaling pathway, Wnt signaling pathway, MAPK signaling pathway, ErbB signaling pathway etc. (see Additional file 4).

### Variation frequency evaluation

Table 8 illustrates the counts of driver genes with low or high variation frequency identified by ours and other six methods for each cancer type. The identified driver genes with low variation frequency are those mutated in equal to or less than three samples, others are driver genes with high variation frequency. The top of Table 8 lists the number of real driver genes with low variation frequency detected by each method when selecting top 50, 100, 150 and 200 genes as candidates. The result shows our method can figure out some driver genes with low variation frequency. Although, it cannot say our model is superior than others, the gap is very small or even zero.

**Table 8:** Number of driver genes with low or high variation frequency identified by our method and six other existing methods

		Top genes	Ours	Diffusion	DriverNet	Muf_max	Muf_sum	IntDriver	DawnRank
Low Frequency	Breast	50	0	1	0	0	1	0	0
		100	0	2	0	0	2	0	0
		150	0	2	0	0	2	0	0
		200	2	2	1	0	4	0	1
	Lung	50	0	0	1	0	0	0	0
		100	0	3	1	0	2	0	2
		150	1	3	2	2	2	0	4
		200	1	4	2	7	2	0	5
	Prostate	50	3	4	2	2	2	2	1
		100	3	4	4	2	2	3	1
		150	3	5	3	6	2	3	1
		200	3	7	5	6	2	3	2
High Frequency	Breast	50	25	11	17	13	13	13	16
		100	32	26	29	20	21	20	22
		150	40	33	39	26	29	26	28
		200	43	41	40	27	38	31	34
	Lung	50	21	15	15	9	11	8	10
		100	32	22	23	12	19	16	19
		150	39	26	27	12	27	20	22
		200	43	35	29	12	30	29	25
	Prostate	50	15	6	9	1	12	10	7
		100	20	8	14	1	16	11	9
		150	21	10	16	10	19	12	11
		200	23	13	17	10	20	14	13

The table shows the number of driver genes identified by our method and other six methods with low variation frequency (mutated less or equal to three samples) or high variation frequency (mutated more than three samples) for each cancer type

Besides, we also verify the capability of our method in identifying driver genes with high variation frequency (*>* 3 samples) by comparing with other six methods. The bottom of Table 8 shows that our capability of identifying driver genes with high variation frequency in all cancer types is obviously superior to other six methods.

Above results indicate that although the variation frequency is involved in our method, it does not weaken our capability in identifying driver genes with low variation frequency because our method introduces gene functional network information for prediction. On the contrary, adding variation frequency enhances our capability in identifying the driver genes with high variation frequency.

### Robust analysis

**Fig. 5:**
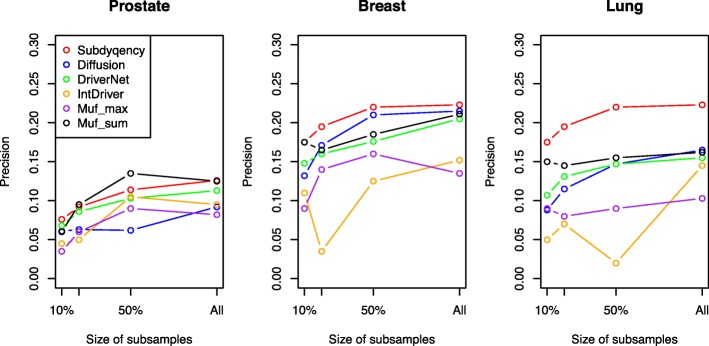
A comparison of robustness on three cancer datasets. The X-axis represents the percent of total number of sample size The Y-axis represents the corresponding precision value of each method when select part of samples for prediction

The final result may be impacted by the quantity of discussed samples due to the variation frequency that is calculated based on the total sample size for each cancer type. Hence, to validate the robustness of our method, at first, we randomly generated a series of sample subsets with different sizes 10, 20, 50% of the total number patients for each cancer type. Then, we applied our algorithm on each subset and repeated the process 10 times. The whole test process is similar to the Shi’s diffusion algorithm [17]. Figure 5 shows the average precision of ours and other five methods when selecting top 200 identified genes. Since there are limited number of samples that both have normal and cancer expression profiles, we do not include the DawnRank in this test. It can be seen that the precision values decrease significantly when the sample size is smaller than the 50% of total counts, while, keeping in a relatively steady status after that. Even if the sample sizes changed, our method is still superior other methods in breast and lung cancers. Our precision of prostate is similar or slightly lower than that of the Muf_sum. The precision values are 0.076, 0.092, 0.114, 0.126 for ours and 0.06, 0.095, 0.135, 0.125 for Muf_sum in 10, 20, 50% and all of sample sizes. Maybe, this is because only 236 driver genes can be found from the NCG 4.0 and meanwhile, we chose the top 200 candidate driver genes to evaluate. Hence, the difference between our method and Muf_sum is not so obviously. However, our results in prostate are still better than other six methods.

In summary, the result shows even with a small subset of patients, our method in general is better than other methods. Hence, it can be said that Subdyquency is robustness enough to adjust different sample sizes.

## Discussion

In this paper, we have proposed a method called Subdyquency to identify the cancer driver genes. We assumed that driver genes are more likely to regulate the downstream gene’s expression in the same compartment and the interaction in the significance compartment is more reliability than that in the lower importance compartment. Hence, the Subdyquency incorporates with mutated genes’ own profile (variation frequency) and its interactions with other dysregulated genes in a certain compartment (subcellular localization). The result shows that our model can achieve a higher performance in precision, recall and fscore aspects than other six methods. The interesting and novel finding is that some new unknown potential driver genes which are co-cited by other literatures also can be found by Subdyquency. Besides, our results enrich in some significant cancer pathways and GO functions.

In the future, we hope to improve the performance of our method to a higher level by filtering the variation frequency based on the DNA length. Because the longer the genes are, the more chance of them to be the mutated genes [31]. Besides, we want to construct a new interaction network among mutated genes involving other cancer-related profiles such as the tissue-specific profile. We also want to consider the heterogeneous between different cancer types in order to deeply improve the performance for some specific cancers.

## Conclusions

In recent years, there are many methods and tools have been proposed to identify driver genes. However, they still have some limitations such as low precision and fail to comprehensively consider both the biological properties and the network topological properties of driver genes. In this study, we developed a new method by integrating mutated genes’ own profile (variation frequency) and its interactions with other dysregulated genes in a certain compartment (subcellular localization) to pinpoint the candidate driver genes. We set the parameter *α* to coordinate the importance of variation frequency and interactions. According to the AUC values when setting *α* to different values, we assigned *α* with 0.5 which means the same importance between mutated genes’ own profile and its interactions network. We applied our method on three different cancers (lung, prostate, breast) and compared the results with other six existing methods (DriverNet, Diffusion, Muf_max, Muf_sum, DawnRank, IntDriver). The prediction of known cancer genes shows our method is superior to other six models in terms of precision, recall and fscore. The literature mining results indicate our method can not only prioritizes some significant driver genes but also recognizes the rare unknown driver genes with high co-cited counts. Furthermore, the functional enrichment analysis shows that the driver genes identified by our method enrich in some important functions and some cancer related significant pathways. The analysis on prediction results with respect to different variation frequency displays our method has capability to prioritize driver genes regardless of it is low variation frequency (mutated equal or less than 3 samples) or high variation frequency (mutated more than 3 samples). Unlike previous computational based methods, our method stands at the biological perspective to hypothesize that the driver genes mutate in many samples and impact more downstream genes in the common compartment.

## Additional files

Additional file 1: The results of prediction of known cancer genes for lung, breast and prostate cancer. (XLSX 13 kb)

Additional file 2: The results of GO and KEGG enrichment analysis in lung cancer. (XLSX 114 kb)

Additional file 3: The results of GO and KEGG enrichment analysis in breast cancer. (XLSX 147 kb)

Additional file 4: The results of GO and KEGG enrichment analysis in prostate cancer. (XLSX 112 kb)

## Abbreviations

BRCA: Breast invasive carcinoma
LUAD: Lung adenocarcinoma
Muf_max: Muffinne_max
Muf_sum: Muffinne_sum
PPI: Protein to protein interaction network
PRAD: Prostate adenocarcinoma
